# No evidence for after-effects of noisy galvanic vestibular stimulation on motion perception

**DOI:** 10.1038/s41598-020-59374-9

**Published:** 2020-02-13

**Authors:** Aram Keywan, Hiba Badarna, Klaus Jahn, Max Wuehr

**Affiliations:** 1 German Center for Vertigo and Balance Disorders, Ludwig-Maximilians University of Munich, University Hospital Grosshadern, Munich, Germany; 2 Schoen Clinic Bad Aibling, Department of Neurology, Bad Aibling, Germany

**Keywords:** Sensory processing, Translational research

## Abstract

Noisy galvanic vestibular stimulation (nGVS) delivered at imperceptible intensities can improve vestibular function in health and disease. Here we evaluated whether nGVS effects on vestibular function are only present during active stimulation or may exhibit relevant post-stimulation after-effects. Initially, nGVS amplitudes that optimally improve posture were determined in 13 healthy subjects. Subsequently, effects of optimal nGVS amplitudes on vestibular roll-tilt direction recognition thresholds (DRT) were examined during active and sham nGVS. Ten of 13 subjects exhibited reduced DRTs during active nGVS compared to sham stimulation (p < 0.001). These 10 participants were then administered to 30 mins of active nGVS treatment while being allowed to move freely. Immediately post-treatment , DRTs were increased again (p = 0.044), reverting to baseline threshold levels (i.e. were comparable to the sham nGVS thresholds), and remained stable in a follow-up assessment after 30 min. After three weeks, participants returned for a follow-up experiment to control for learning effects, in which DRTs were measured during and immediately after 30 min application of sham nGVS. DRTs during both assessments did not differ from baseline level. These findings indicate that nGVS does not induce distinct post-stimulation effects on vestibular motion perception and favor the development of a wearable technology that continuously delivers nGVS to patients in order to enhance vestibular function.

## Introduction

There is growing evidence that information processing in sensory systems can be enhanced by adding an appropriate low-intensity level of noise to the system^1–4^. The rationale behind this phenomenon is a mechanism known as stochastic resonance (SR), according to which the response of a non-linear system to weak input signals can be optimized by the presence of a particular level of stochastic interference, i.e., noise^5,6^. SR-like phenomena in the vestibular system can be induced by an imperceptible noisy galvanic vestibular stimulation (nGVS), which was shown to effectively lower thresholds for vestibular motion perception and vestibulospinal reflexes^7–11^. The therapeutic potential of nGVS was further explored in patients with vestibular hypofunction that suffer from pathologically increased thresholds for vestibular information processing^12,13^. nGVS-treatment in these patients resulted in improved static postural and dynamic gait stability^14–17^.

With respect to future therapeutic applications of nGVS, it is important to determine whether nGVS-induced improvements in vestibular information processing are only present during active stimulation or will sustain after a prolonged treatment with this stimulation. In line with the latter possibility, several studies on supra-threshold GVS could demonstrate profound after-effects on the ocular-motor and postural domains after stimulus termination^18–23^. Furthermore, two recent studies suggest that also low-intensity, sub-threshold nGVS can induce sustained post-stimulation improvements in body balance in healthy elderly and in patients with bilateral vestibular hypofunction^24,25^. These findings are, however, at variance with the presumed mechanism of nGVS, where SR requires the simultaneous presence and interaction of sub-threshold incoming signals and low-intensity noise. Nevertheless, it is still possible that SR-induced peripheral or central vestibular adaption processes might result in a sustained improvement of vestibular function after the cessation of the stimulus. Both studies, however, did not control for possible placebo and/or task-dependent learning effects, which in turn makes it difficult to ascertain the origin of the reported improvements.

The aim of the present study was therefore to re-examine the possibility of sustained after-effects of nGVS on vestibular function, in particular on vestibular motion perception. The latter has been recently demonstrated to be enhanced by the same nGVS amplitudes that also improve posture^7,11^. We thus studied and compared the effects of nGVS when actively applied during a vestibular direction-recognition task, to its effects on the same modality following a 30-min nGVS treatment while freely moving. The experimental setup was further controlled for possible cofounding placebo and task-dependent learning effects.

## Methods

### Ethics

The study protocol was approved by the ethics committee of the medical faculty of the Ludwig-Maximilians University of Munich (reference number: 496-16). The study was conducted in conformity with the Declaration of Helsinki. Informed written consent was obtained from all participants prior to the experiments.

### nGVS stimulation

**Figure 1:**
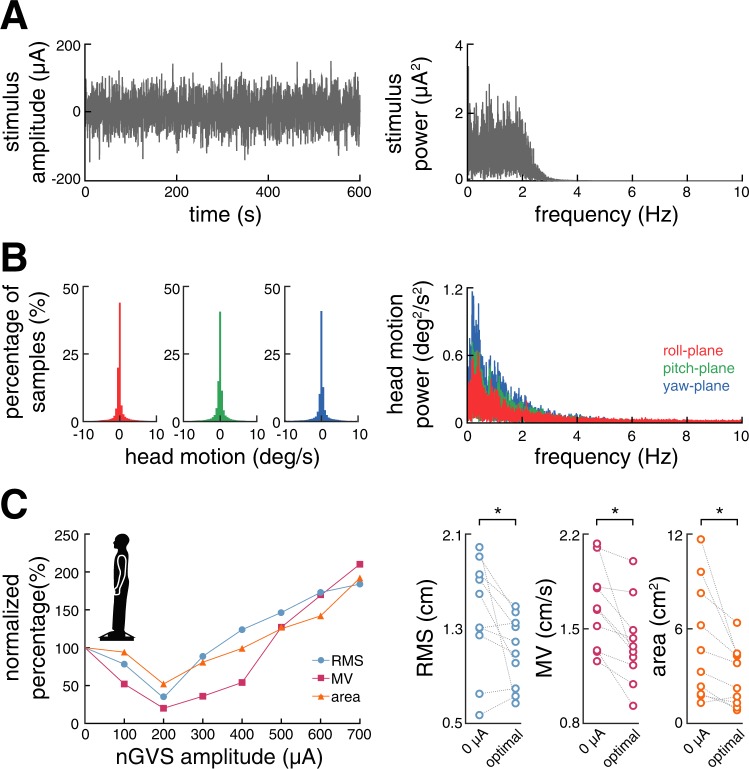
nGVS stimulus characteristics, head motion profiles, and nGVS effects on posture. (**A**) Exemplary nGVS stimulus profile (left panel) and corresponding nGVS power spectrum (right panel). (**B**) Distribution of head angular velocity magnitudes (bin width: 0.5 deg/s) of one exemplary participant during the 30 min nGVS stimulation between session 2 and 3 (left panel) and corresponding head motion power spectra (right panel). (**C**) Exemplary balance responses to nGVS at varying amplitudes that follow a bell-shaped curve with maximal improvement at 200 µA (left panel). Corresponding group effects of nGVS at 0 µA (i.e., baseline) compared to nGVS at optimal intensities. *Indicates a significant difference between conditions.

Thirteen healthy subjects (four males; mean age 25.6 ± 2.8 years) participated in the study. The sample size was calculated with respect to effect sizes reported in previous studies on nGVS effects on vestibular motion perception^7,8,11^, with a power of 0.80, an alpha level of 0.05, and an effect size of 0.75. nGVS in each participant was applied via a pair of 4.0 cm × 6.0 cm Ag-AgCl electrodes attached bilaterally over the left and right mastoid process. A constant current stimulator (DS5, Digitimer, Hertfordshire, UK) delivered a zero-mean Gaussian white noise within a frequency range of 0–2 Hz (Fig. 1A)^7^. All participants were familiar with the experimental procedure from a previous study^7^.

### Identification of optimal nGVS amplitude

Body sway of each subject was recorded for 30 s by a stabilometer platform (Kistler 9261 A, Kistler Group, Winterthur, Switzerland) while standing on foam with eyes closed. This procedure was repeated eight times, each time with different amplitude of nGVS, ranging from 0–700 µA in a randomized manner. For each trial, three parameters characterizing body sway were analyzed offline: The mean velocity (MV) and root mean square (RMS) of the center of pressure (COP) in the anterior-posterior (AP) and medio-lateral (ML) planes as well as the envelopment area traced by the COP movement. The ratio of each parameter during the stimulation conditions to that of the baseline condition (i.e., 0 µA) was calculated (i.e., normalized ratio). The nGVS amplitude that caused the greatest reduction in the normalized ratios of all three stance parameters (i.e., enhanced postural control) was determined as the optimal nGVS amplitude. Between trials, subjects had a 1 min break.

### Vestibular threshold determination

After ascertaining the optimal nGVS amplitude, subjects performed direction-recognition experiments in the roll-plane at 1 Hz (150 trials each, 3-down 1-up paradigm) using a 6-degree-of-freedom motion platform (Moog 6DOF2000E, East Aurora, New York). Each trial consisted of a single half-cycle that followed a raised-cosine velocity profile to the right or to the left^7,26,27^ and subjects had to indicate the direction of movement by a button press. A cumulative Gaussian psychometric curve was then fitted to the response data. The direction recognition threshold (DRT), which corresponds to our performance metric, is the magnitude of roll-tilt that can be correctly distinguished at a rate of 79.4%^28^. Direction-recognition tasks were used to minimize the influence of vibration and other non-directional cues on vestibulo-perceptual thresholds^29^. Noise-cancelling head-phones were used to mask incoming sound cues from the platform. All experiments were performed in total darkness with eyes closed.

### Procedures

**Figure 2:**
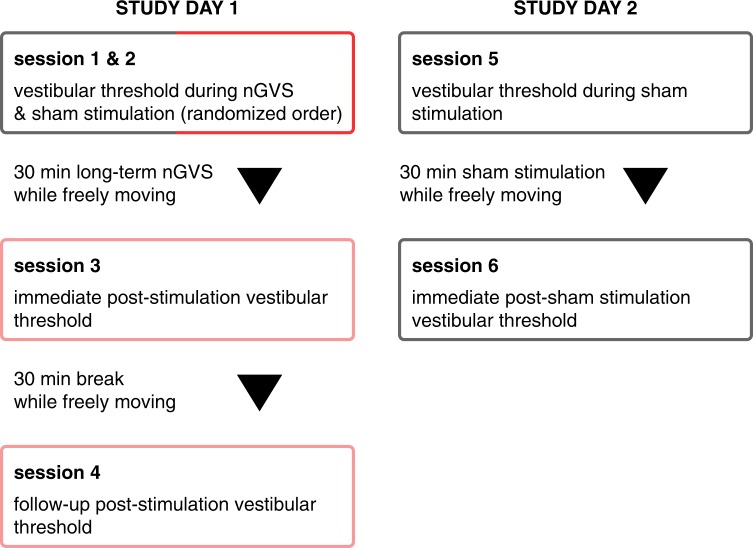
Flowchart of the experimental procedures. The experimental protocol consisted of six threshold determination sessions conducted over two study days with a three week break in between. Between sessions, participants were administered to once a 30 min period of nGVS, a 30 min period of sham nGVS stimulation and a 30 min period without any stimulation. During these periods, participants were allowed to freely move in the lab.

The experimental procedures consisted of six separate threshold determination sessions conducted on two different days, with a-three week break in between (Fig. 2). On study day 1, each participant initially performed two threshold determination sessions, once during non-zero nGVS (optimal amplitude) and once during sham stimulation (0 µA) in a randomized order (Fig. 2, session 1 & 2). Subjects in whom nGVS induced an enhancing effect on motion perception (n = 10), were enrolled to the subsequent experiments. Those who did not (n = 3), were excluded from further assessments. The remaining 10 participants were then exposed to nGVS stimulation for 30 min whilst freely moving in the lab (i.e., sitting, standing or walking). Head kinematics during this free-moving interval were monitored in the first two subjects using a head-fixed inertial sensor (EyeSeeCam, Munich, Germany) (Fig. 1B). Following this 30 min stimulation, subjects immediately repeated a threshold determination session to examine any post-stimulation effects on vestibular motion perception (session 3). After a subsequent 30 min interval during which subjects could again freely move in the lab without any stimulation, a follow-up threshold determination session was performed to examine whether any sustained effects of the stimulation could be observed (session 4). On study day 2, three weeks after the initial assessment, each participant returned for a control experiment. To control for possible placebo and/or task-dependent learning-effect, subjects performed a sham nGVS threshold determination session (session 5), which was followed by a 30 min sham stimulation (starting with a supra-threshold level for 30 s, which gradually decayed to 0 mA over another 30 s) whilst freely moving in the lab. Subsequently, subjects underwent a final post-sham threshold determination session (session 6).

### Statistical analysis

Data are reported as mean ± SD. Statistical analysis was performed on participants who showed nGVS-induced vestibular threshold reduction compared to sham stimulation (n = 10). Analyses were conducted on the log-transformed DRTs to achieve normal distribution^26,30,31^, which was confirmed by a Shapiro-Wilk-Test. Using a repeated-measures ANOVA and Bonferroni post hoc analysis, effects on body sway were analyzed with trial (baseline vs. optimal trial) as factor and effects on DRTs were analyzed with session (four sessions on study day 1 and two sessions on study day 2) as factor. Results were considered significant if p < 0.05. Statistical analysis was performed using SPSS (version 21.0, IBM Corp., USA).

## Results

**Figure 3:**
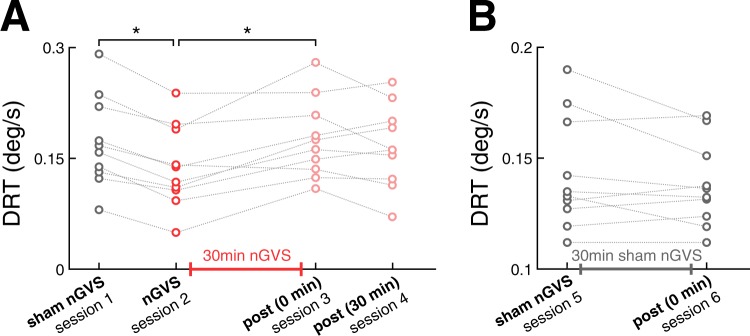
Active- and post-stimulation effects of nGVS on vestibular motion perception thresholds. (**A**) Baseline direction recognition thresholds (DRTs, session 1) as well as active- (session 2) and post-stimulation (session 3 & 4) effects of nGVS on DRTs on study day 1. Session 1 and 2 were conducted in randomized order. (**B**) Effects of active- (session 5) and post-stimulation (session 6) sham nGVS on DRTs on study day 2. *Indicates a significant difference between conditions.

For all participants, we found optimal nGVS amplitudes (220 ± 155 µA, range 100–600 µA) that effectively improved posture compared to baseline performance (Fig. 1C; RMS: F_1,9_ = 5.4, η^2^_*p*_ = 0.37, p = 0.046; MV: F_1,9_ = 12.6, η^2^_*p*_ = 0.58, p = 0.006; area: F_1,9_ = 7.6, η^2^_*p*_ = 0.46, p = 0.023). Ten out of 13 subjects showed improved DRTs during the application of nGVS at optimal amplitude compared to sham stimulation (session 1 & 2). In these 10 participants, nGVS decreased DRTs by an average of 21.0 ± 8.8% (F_5,45_ = 5.6, η^2^_*p*_ = 0.38, p < 0.001 Bonferroni adjusted). Subsequently hereafter, participants were administered to a 30 min application of nGVS at optimal amplitude during which they were allowed to freely move in the lab. Exemplary head motion tracking in two participants during this period revealed that the predominant head velocity frequency fell within the nGVS frequency bandwidth (0–2 Hz) and that a considerable amount of vestibular inputs remained below commonly reported detection thresholds of vestibular afferents (Fig. 1)^32^. However, immediately following the application of a prolonged nGVS stimulation, DRTs had returned to baseline level, i.e., were comparable to the initial threshold determined during sham stimulation and significantly higher than the thresholds obtained during active non-zero nGVS application (session 3; F_5,45_ = 5.6, η^2^_*p*_ = 0.38, p = 0.044 Bonferroni adjusted). A follow-up vestibular threshold test after a subsequent break of 30 min, demonstrated no further changes in DRTs (session 4; Fig. 3A).

On study day 2, after three weeks, participants repeated the vestibular threshold determination twice, with a 30 min sham stimulation interval in between, in order to control for potential placebo and task-dependent learning effect. DRTs determined during sham stimulation (session 5) and immediately after prolonged sham stimulation (session 6) did not differ from the baseline DRTs obtained during study day 1 (Fig. 3B).

## Discussion

In this study we examined possible post-stimulation effects of nGVS delivered at imperceptible intensities on vestibular motion perception thresholds. We observed that those study participants who positively responded to nGVS in terms of enhanced vestibular motion perception during the thresholding task, did not exhibit any measurable after-effects on motion perception following a 30 min nGVS treatment while freely moving. We also controlled for possible placebo and task-dependent learning effects and found that moderate task-dependent learning was present, yet its magnitude was not significant.

Several previous studies demonstrated that sub-threshold as well as supra-threshold GVS can have immediate facilitatory effects on various perceptual conditions and modalities, such as hemi-spatial neglect^33^, spatial memory^34^, verticality perception^18^, subjective visual and tactile vertical^35^, and face recognition^36^. In the case of a prolonged supra-threshold application of GVS, there is broad evidence for a measurable sustained effect after stimulus termination. Immediately after cessation of a prolonged application of supra-threshold GVS, individuals will commonly experience self-motion perceptions, postural, and ocular-motor responses of a similar magnitude as during stimulation but oppositely directed^18–22^. These after-effects have been attributed to central adaptation processes to the prolonged artificial vestibular stimulus^19^.

On the other hand, sub-threshold nGVS is thought to exert its effect on vestibular information processing by the mechanism of SR. According to this mechanism, weak incoming vestibular signals get amplified by interacting with the low-intensity noise stimulus and thereby become detectable^5,6^. Hence, this mechanism requires the mutual presence of a weak, sub-threshold input signal as well as low-intensity noise. Therefore, theoretically, noise-induced alterations in vestibular information processing should immediately disappear after nGVS termination, which is in line with the present findings. The presumed working principle of nGVS together with the current results are, however, at variance with two recent studies by Fujimoto and colleagues, who reported sustained after-effects of nGVS on postural stability of healthy elderly and patients with bilateral vestibulopathy^24,25^. Although it is possible that these after-effects might reflect SR-induced peripheral or central vestibular adaption processes, there is no experimental evidence in these studies to support such a mechanism. In particular, these studies did not control for task-dependent learning effects, which are known to occur in the course of repeated posturographic examinations^37,38^. In fact, Maheu and colleagues recently re-examined the presence of after-effects of nGVS on postural performance in a placebo-controlled experimental setup and did not find any lasting post-stimulation effects of nGVS on postural stability when compared to sham stimulation^39^.

Nevertheless, the lack of evidence for nGVS after-effects on vestibular motion perception cannot completely exclude potential sustained effects of nGVS on other vestibular functions, such as balance-control via vestibulospinal pathways. However, nGVS is thought to primarily affect thresholds for peripheral vestibular information processing and should therefore equally influence vestibulo-perceptual and vestibulo-spinal pathways. Previous studies, which showed that nGVS delivered at the same amplitudes that stabilized posture also improved vestibular motion perception^7,11^ support this notion. Furthermore, the present stimulation protocol differed from the previous studies on sustained nGVS-effects on posture in terms of a narrower frequency band of the nGVS stimulus (0–2 Hz vs. 0–10 Hz). However, Mulavara and colleagues demonstrated that the effects of narrow and wideband nGVS stimuli on posture are comparable^40^. Finally, the sample size in the current study was only powered to detect large effects between stimulation conditions, meaning that smaller after-effects of nGVS might have gone undetected.

Recently, nGVS has been proposed as a potential future treatment option for rehabilitation in patients with vestibular hypofunction^16^. It is, however, not known in what form (continuous vs. intermittent stimulation) nGVS should be applied to optimize treatment effects and ensure patients’ tolerability to treatment. The here observed absence of sustained stimulation after-effects on vestibular performance has to be considered for treatment protocols. It suggests that nGVS might be suitable for an intermittent treatment only when a carry-over of improvements achieved during stimulation is ensured by the training protocol. Effective treatment approaches with nGVS might also require a long-term continuous application of vestibular stimulation delivered by a wearable technology. Given the non-invasive and sub-threshold nature of nGVS, it is reasonable to be well tolerated by patients^41,42^.

## Data Availability

The datasets generated during and/or analyzed during the current study are available from the corresponding author on reasonable request.
